# Demand sensing and digital tracking for maternal child health (MCH) in Uganda: a pilot study for ‘E+TRA health’

**DOI:** 10.1186/s12911-022-01982-8

**Published:** 2022-09-12

**Authors:** Dawei Wang, Rhoann Kerh, Sungbum Jun, Seokcheon Lee, Roy William Mayega, Julius Ssentongo, Andualem Oumer, Md Haque, Priyanka Brunese, Yuehwern Yih

**Affiliations:** 1 School of Industrial Engineering, Purdue University, West Lafayette, IN 47907 USA; 2 ResilientAfrica Network (RAN), School of Public Health, Makerere University, Kampala, Uganda; 3 Management Science for Health, Medford, MA 02155 USA; 4 R B Annis School of Engineering, University of Indianapolis, Indianapolis, IN 46227 USA; 5 LASER PULSE (Long-Term Assistance and SErvices for Research, Partners for University-Led Solutions Engine) Consortium, Purdue University, West Lafayette, IN 47907 USA; 6 Regenstrief Center for Healthcare Engineering, Purdue University, West Lafayette, IN 47907 USA; 7 Health Economic and Decision Sciences, Merck & Co., Inc., Kenilworth, NJ 07033 USA; 8 Department of Epidemiology & Biostatistics, School of Public Health, Makerere University, Kampala, Uganda; 9 Department of Industrial & Systems Engineering, Dongguk University, Seoul, Republic of Korea

**Keywords:** Demand sensing, Healthcare supply chain management, Maternal child health (MCH), Electronic medical record (EMR)

## Abstract

**Background:**

Thirteen essential maternal child health (MCH) commodities, identified by the UN Commission on Life-Saving Commodities for Women and Children, could save the lives of more than 6 million women and children in Low-and-Middle-Income Countries (LMICs) if made available at the point of care. To reduce stockout of those commodities and improve the health supply chains in LMICs, the Electronic TRAcking system for healthcare commodities (E+TRA Health), an all-in-one out-of-box solution, was developed to track and manage medical commodities at lower-level health facilities in rural areas. It aims to support real-time monitoring and decision-making to (1) reduce the time needed to prepare orders, (2) reduce stockout and overstock cases of targeted medical supplies, (3) help improve patient outcomes. In this study, we adopted an integrated approach to analyze the process of information flow, identify and address critical paths of essential supplies associated with maternal health in the Ugandan health system.

**Methods:**

We apply system engineering principles and work with community partners in hospitals to develop care process workflow charts (based on essential services) for the lifecycle of maternal health continuum of care. Based on this chart, we develop a cloud-based offline-compatible smart sync platform named “E+TRA Health” to triangulate (1) patient admission, diagnoses, delivery information, testing reports from laboratories, (2) inventory information from main store, stores in MCH unit, and (3) lab, to identify the critical list of medical and laboratory supplies, their lead times for procurement and then generate reports and suggested procurement plans for real time decision-making.

**Results:**

The E+TRA Health platform was piloted in two Healthcare Center IV facilities in Uganda over a period of 6 months. The system collected more than 5000 patient records and managed more than 500 types of medicines. The pilot study demonstrated the functionalities of E+TRA Health and its feasibility to sense demand from point of care.

**Conclusion:**

E+TRA Health is the first to triangulate supply and demand data from three different departments (main store, lab, and MCH) to forecast and generate orders automatically to meet patient demands. It is capable of generating reports required by Ministry of Health in real time compared to one-week lead-time using paper-based systems. This prompts frontline stakeholders to generate efficient, reliable and sustainable strategic healthcare plans with real time data. This system improves patient outcomes through better commodity availability by sensing true patient demands.

**Supplementary Information:**

The online version contains supplementary material available at 10.1186/s12911-022-01982-8.

## Introduction

Every two minutes, a preventable death occurs during childbirth in Low-and-Middle-Income countries (LMICs). Complications from pregnancy and childbirth are the leading cause of death among girls aged from 15 to 19 [1, 2]. Inadequate inventory and supplies contribute significantly to preventable death in LMIC settings [1, 3–5]. UN Commission on Life-Saving Commodities for Women and Children identified and endorsed an initial list of 13 overlooked life-saving commodities that could save the lives of more than 6 million women and children, if more widely accessed and properly used [6]. Bill and Melinda Gates Foundation targeted this area in the Round 19 Global Grand Challenges^1^ to ensure effective health supply chains in LMICs. This paper is based on the project selected to develop and pilot a digital solution (E+TRA Health) to strengthen the supply chains of medical commodities to support maternal child health (MCH) in Uganda.

### Healthcare delivery systems

Information of Healthcare delivery is usually captured in two independent systems: health information system and healthcare supply chain management system. Health information system is typically known as the electronic medical record system (EMR), and collects health information about the patients, such as medical history. While, healthcare supply chain management system logs the dispensing of healthcare commodities, monitors inventory levels and restocks? replenishments. The following subsection presents the health information and health supply chain management systems in the context of the Ugandan health system.

#### Health information system in Uganda

Uganda health governance is divided into regions, districts, sub-districts, health facilities, and villages [7]. Accordingly, the health services are structured into National Referral Hospitals (NRHs) and Regional Referral Hospitals (RRHs), General Hospitals, Health Center (HC) IVs, HC IIIs, HC IIs and Village Health Teams (HC Is). At the highest level, NRHs, RRHs, and General Hospitals provide specialist clinical and comprehensive services. At the secondary district level, HC IIIs offer basic preventive, promotive and curative care. HC IIs only provide outpatient care and community outreach services. At the lowest level, Village Health Teams (VHTs)/HC Is facilitate health promotion, service delivery, and community participation [7].

Uganda’s first Health Information System (HIS) was designed in 1985 to capture and analyze data on communicable and non-communicable diseases [8, 9]. A centralized health management information system (HMIS) was introduced in 1993 that focused on morbidity and mortality reporting, collecting data from health units to the districts and national levels [10, 11]. The HMIS was completely paper-based. Monthly reports were generated from VHTs at the lowest level and submitted to HCs. HCs aggregated and submitted reports to the District Health Office (DHO). DHOs compiled reports and submitted to the Ministry of Health (MoH). This cumbersome monthly reporting process that required health workers lots of time to tally by going through logs often results in inaccurate data [18]. There are also other HIS tools, such as “mTrack”, a SMS-based HMIS tool designed to report on disease surveillance, “WinSenga”, a fetal heart rate monitor using smartphones (ibid), etc. Various HIS interventions emerged in Uganda but often ended in pilot phases due to lack of clear evaluation or limited skills, inadequate policy and low adoption by health workers [9].

Uganda’s health system recognizes the benefits of electronic medical record (EMR) systems [12–15], mobile health [16, 17], and risk surveillance systems [18, 19]. However, limited infrastructure and resources hinder the design and implementation of these systems. In addition, current EMR systems in Uganda scarcely address the needs of MCH or primary care, mostly focusing on specifically communicable diseases such as HIV, malaria, and tuberculosis. (i.e., Uamuzi Bora Kenya [20], PIH-EMR Peru [21], HIV-EMR Haiti [22], OpenMRS Uganda [23], Lilongwe EMR Malawi [24]).

A system to capture the consumption and needs of maternal child health (MCH) commodities at each health facility is needed to guarantee high levels of service and minimize stockouts [25]. Many healthcare systems in Uganda implement standardized data registers to capture patient information and health product inventory status. However, in lower-level health facilities (e.g., Healthcare Center IV), limited computer resources prevent digitizing up-to-date recordkeeping [8]. Consequently, there is no digital data management system to capture information about product consumption and inventory. Staff at these healthcare facilities manually collect information from multiple paper-based registers. The main challenge for these paper registries is that health workers do not have consistent standards in maintaining records which makes accurate data capture impossible and hard to support real time decisions [26, 27]. At the district level, lack of patient registers, stock cards, and lab results brings barriers for stakeholders to make evidence-based commodity orders. Long resupply intervals aggravate stockout and expired medication problems [25]. Lack of coordination and human errors cause delays and waste resources, weakening responsiveness of the healthcare supply chain and putting patients at risk.

#### Healthcare supply management system

Although some EMR systems are increasingly amenable to monitor and integrate maternal and child health services in developing countries [28], few studies have addressed the application of EMR in inventory management [8, 29]. Tracking medicines, supplies and lab reagents in developing countries including Uganda is still paper-based stock books/stock cards/dispensing logs. This analog and manual tracking leads to difficulty in recording transactions in real time and requires tremendous effort and time to compile information. Duplications and errors of information make it difficult for store managers to prepare accurate consumption reports to generate right orders. Also, lack of coordination between MCH units and the main store causes difficulties in future demand/order forecasting [30, 31]. This results in stockout or overstock issues, which jeopardize the access to specific medical supplies, thus impacting survival and safety of pregnant women and their newborns. Therefore, there is a need for a digital healthcare supply management system that is tailored to meet MCH patient needs. Thus, in resource limited settings like Uganda, evidence based, coordinated, accurate stock management and quantification can prevent dangerous stockouts of health products. Also, precise ordering requires integration of quantification, EMR, dispensing and inventory control.

Several computerized systems need to be combined for a healthcare supply chain system *EMR* systems record patient history, medicine regimens and dosages. *Dispensing* systems record health products dispensed (i.e., mSupply, iDart, RxSolution, ADT). *Inventory control* systems track individual supplies by names, batch numbers, stock quantities and expiration dates (i.e., SIGMED, ORION, mSupply, HIV-EMR Pharmacy system, Navision, Syspro, ePICs). *Quantification* systems assist in calculating budget requirements and order quantities (i.e., FoCaMed, Quantimed, RxSolution, PIH-EMR, MSF ARV Drug Order Tool). However, EMR, Inventory control, and Dispensing systems manage patient data, inventory data and dispensing data separately. Moreover, all these tools have been mainly applied at the national and/or regional/state levels, not at the clinical level. Therefore, there is no all-in-one solution that has the capability to integrate all demand and supply data together to suggest a procurement plan at the clinical level.

Despite substantial needs, very few software applications are available in resource scarce environments. Current limitations include: (1) none of the existing systems combine the EMR, inventory control, dispensing and quantification systems. (2) Not all datasets are considered when preparing order quantities, which diminishes accuracy of order data due to the lack of data accessibility. (3) Most application designs are complex and geared toward national warehouses or retail pharmacies. (4) Most systems are proprietary, making customization and technical support difficult and costly. (5) Systems are designed under the assumption that high-bandwidth internet networks are available. (6) Supplies for test and laboratory regiments for preventive/diagnostic care are often not linked to the patient demands.

The focus of this study is to analyze information flow and design a health information technology solution to address gaps in the last mile supply chain associated with MCH in the Ugandan health system.

## Methods

Healthcare information systems struggle in implementation in LMIC settings due to limited local infrastructure, resources, and capacities. Many systems require high-bandwidth connections. The system we proposed is an all-in-one solution that can be used without access to the Internet. We created a local network that can exchange information within the facility. The system is a local cloud-based system coded in PHP, HTML, JavaScript, and CSS backed with SQL databases.

### Step 1: site selection

The study was conducted in Mukono district, Uganda. The district is located in the central region of Uganda, with a population of 596,804 people (UBOS, 2014). Mukono district has a total of 51 health facilities that are points of delivery for primary health care, and these include: one general hospital, three health center IVs, 15 health center IIIs, and 32 health center IIs. Our study was conducted in two health center IVs, namely Mukono and Kojja. We selected these two health facilities as they represent primary health care seeking behaviors and other dynamics at both urban/peri-urban and rural settings. Mukono HC IV represents an urban public health facility setting and it conducts over 500 deliveries per month. On the other hand, Kojja HC IV is situated in a rural setting which is similar to many of Uganda’s PHC facilities. It conducts about 100 deliveries per month.

### Step 2: understanding issues facing health practitioners in selected sites

This study used an iterative co-design process among academics, practitioners, and other stakeholders. It has similar characteristics to Integrated Knowledge Translation (IKT), a collaborative research approach involving health practitioners as equal partners alongside researchers, with the goal of creating more relevant and useful solutions that result in better research outcomes [32–35]. The key principle of IKT is involving practitioners throughout the research process starting with identification of the research question, and are actively engaged in the governance, priority setting and conduct of the research [32].

IKT was established by the Canadian Institute of Health Research (CIHR) and has evolved from traditional ‘Knowledge Translation’ approaches which goes beyond the reductionist view of knowledge translation that typically involves ‘translating’ research findings at the end-of-the-grant research by filling the gap with ‘translated products for dissemination’; to co-producing knowledge for ‘actionable evidence’. IKT has been applied specifically in public health intervention research [36] in Canada, with the assumption that it can increase the uptake of research evidence into policy and practice as the collaboration process between researchers and practitioners will generate knowledge that is relevant to practitioners [32, 37, 38].

In this project, partners are integrated early and throughout the project for solutions more likely to be adopted and applied. Researchers including a team from Makerere University Resilient Africa Network (RAN) engaged and partnered with the health providers at the Kojja Health Center IV, the Mukono District Health Office, and the Mukono Health Center IV in Uganda. To ensure a solid foundation to work together effectively, meetings were conducted to establish collaboration and to understand the specific needs of the key stakeholders. The solution was co-designed between the research team and care providers and implemented in two health centers. by including the end users of the system in identifying and prioritizing the requirements. Through this collaboration, the research team was able to understand the workflow for patient care and existing data management systems in the local facilities.

For example, the team learned that it required 2–3 days to prepare a bi-monthly order and a week to prepare a monthly report required by MOH in Uganda. There was duplicate data recorded in different hand-written registries. The order quantities for medical supplies were estimated based on existing stock levels instead of future patient needs. The lack of digital data caused issues in commodity management (shortage and overstock) that has direct impacts on patient care. These learnings were used as inputs to the functions and interfaces of the system design.

### Step 3: development of system requirements

The all-in-one healthcare supply chain management system tailored for the MCH unit that we propose needed to triangulate supply chain, patient, lab dispensing data all together to fulfill the following three requirements of each sector listed in Table 1:

**Table 1:** System requirements

Sector	Function	Requirements
	Sourcing	Once the commodities from the national medical store arrive at the healthcare facility, record item details such as names, quantities, expiry dates, arrival times
Supply Chain Management (SCM)	Item coding	Assign unique codes to identify commodities so that users can easily pull-out records for each item for detailed analysis and audits
	Dispatching	Capture quantities of products dispatched to wards such as the MCH unit and the lab. Record logs of dispensed commodities to capture consumption rates
	Inventory management	Capture up-to-date sourcing and dispensing records. Aggregate logs to generate monthly transaction reports. Let warehouse managers manually override and adjust inventory levels and record it with reasons to keep transparency
	Determination of order quantities	Suggest order quantity for each health product. Store managers can refer to the suggested order. Forecasted order quantities generated can be used directly as the order
Electronic Medical Record (EMR)	Patient record	Contains all information about the patient, such as admissions, prescriptions, diagnoses, lab results, antenatal records, and deliveries. Prescriptions are used to calculate consumption rates of health products
	Ministry of Health (MOH) Monthly reports	Generate monthly reports in standardized formats as required by Uganda MOH
Demand Sensing Integration	Lab dispensing log	Besides EMR (patient data) and SCM (inventory data), record daily activities in the labs: number of tests and patients, number not performed due to lack of supplies, and quantity of supplies and reagents used
	Integration	Triangulate consumption and sourcing data from labs, stores, and MCH units with patient data to forecast and generate purchase orders automatically

### Step 4: development of system architecture

**Fig. 1:**
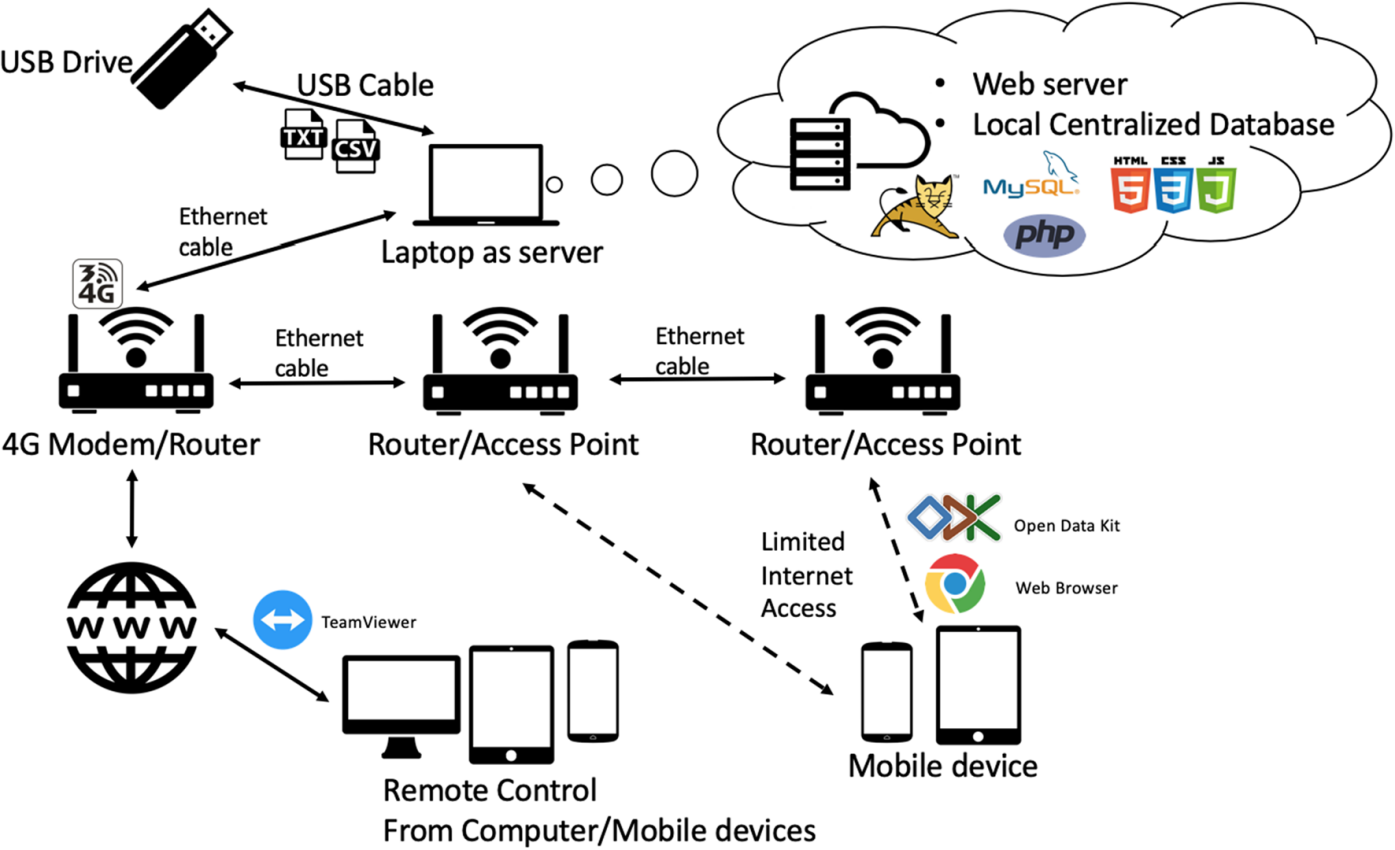
E+TRA health system architecture

The proposed architecture uses a cloud-based centralized database (Fig. 1). Data collection is done on Android tablets using an open-source application, OpenDataKit. Compared to computers/laptops, tablets are cheaper and more portable. They have longer battery lives, lasting more than 10 h. This is critical in places with limited power resources. Multiple tablets can be issued to enter data simultaneously. They are all connected to a local wi-fi network via multiple routers that cover different departments/wards. Data collected on those Android tablets are pushed automatically to the centralized SQL database located in a laptop. The laptop is hosting a web server with SQL database, and an OpenDataKit server using Apache TomCat. Data is then visualized on a website that is accessible from any device connected to the wi-fi network. The routers can be connected to a 4G modem to connect to the Internet, so that we can remotely troubleshoot the system and access reports via TeamViewer or other remote-control applications.

#### Key features of E+TRA health system

Key features of E+TRA Health system are listed in the Table 2 to fulfil the system requirements listed in Table 1.

**Table 2:** Key features of E+TRA health system

Feature	Detail
Cloud-based	Any device with a web browser can use this system, including computers, smart phones, and tablets, with no installation or software updates. All maintenance and updates are done at the server side
Coded in open-source language	The website is coded in an open-source language, PHP, which is relatively easy to develop and maintain. As of 2019, 79% of all server-side websites use PHP [39]. And it is the most-used open-source software within enterprises [40]
Offline-compatible	In developing countries, different departments of a healthcare facility are quite far from each other. Some locations are not covered by wi-fi signals. There are power outages that shut down the routers. Open-source data collection software (e.g., OpenDataKit) provides offline function (Additional file 1: Appendix Fig. S1). Data is stored locally on the devices not covered by wi-fi signals and is uploaded and synchronized automatically when they get access to the local network
Cross-platform	Accessible in different operating systems, e.g., Windows, Mac OS, Android, iOS, etc
transparency	Track any item from receiving from national/district medical stores to dispensing to patients. All transactions/movements and manual adjustments are recorded
Automatic report generation	Generate monthly standardized reports in real time, which are required to submit to the Ministry of Health of Uganda every month, would take one week for staff to manually generate (Additional file 1: Appendix Fig. S2). Visualize data collected (Additional file 1: Appendix Fig. S3) to support decision making
Full patient record	Once admitted during their first visit, future visit histories will be connected automatically via patient ID that is assigned
Automatic inventory level updates	Supply data is extracted from the sourcing forms. Consumption data is extracted from patient prescriptions and lab activities. Store managers no longer manually update and track stock levels on paper or spreadsheets. Full history of transactions of each commodity is recorded in the system and visualized (Additional file 1: Appendix Fig. S4)
Generation of order quantities	Triangulates data collected from MCH, lab, and main store to forecast the order quantities to the national store, based on maximum stock levels of the health facility

### Step 5: integrating system with practitioner workflow

To tailor for the MCH unit, we have reevaluated the needs of users and redesigned forms and users’ workflow.

- *Main store* good receiving note and good delivery note.
  The system was integrated with the ‘main store’ workflow by the development of the ‘notes feature’. The g*ood receiving note* was used to record what items have been received from the national/district medical store, while the g*ood delivery note* was used to record what has been dispatched to the MCH unit and the lab. The main store manager was in charge of completing these two forms. Inventory levels in the main store, lab, and MCH are updated automatically, so the main store manager did not need to manually update the inventory levels.
- *MCH unit*
  The system was integrated with the MCH unit workflow by the development of patient forms such as the *admission form, the diagnosis form, the lab report, the prescription form,* and *the delivery form* replace HMIS form 071 (Antenatal Register) and HMIS form 072 (Integrated Maternity Register) (see Additional file 1: Fig. S5).
  To improve workflow in digital forms, two paper-based forms were separated into five digital forms, since fields are filled at disjoint times: admission, lab result, doctor’s diagnosis, prescription issued, and delivery. All five forms were connected via the patient ID. Therefore, the information was filled exactly and only once. Entire patient history was tracked via patient ID. Inventory levels were deducted automatically upon submission of prescription forms.
- *Lab*
  The system was integrated with the Lab workflow by the development of two forms to estimate commodity usage. The *lab commodity dispensing form* captures dispensing information of lab products. Lab products come in large volume bottles for multiple tests. It is difficult to count how many drops are used during each test. The form was designed to be filled when one countable unit of quantity is used, such as one bottle. The *lab daily activity report* captured the number of tests and patients served daily.

### Step 6: design of information flow

**Fig. 2:**
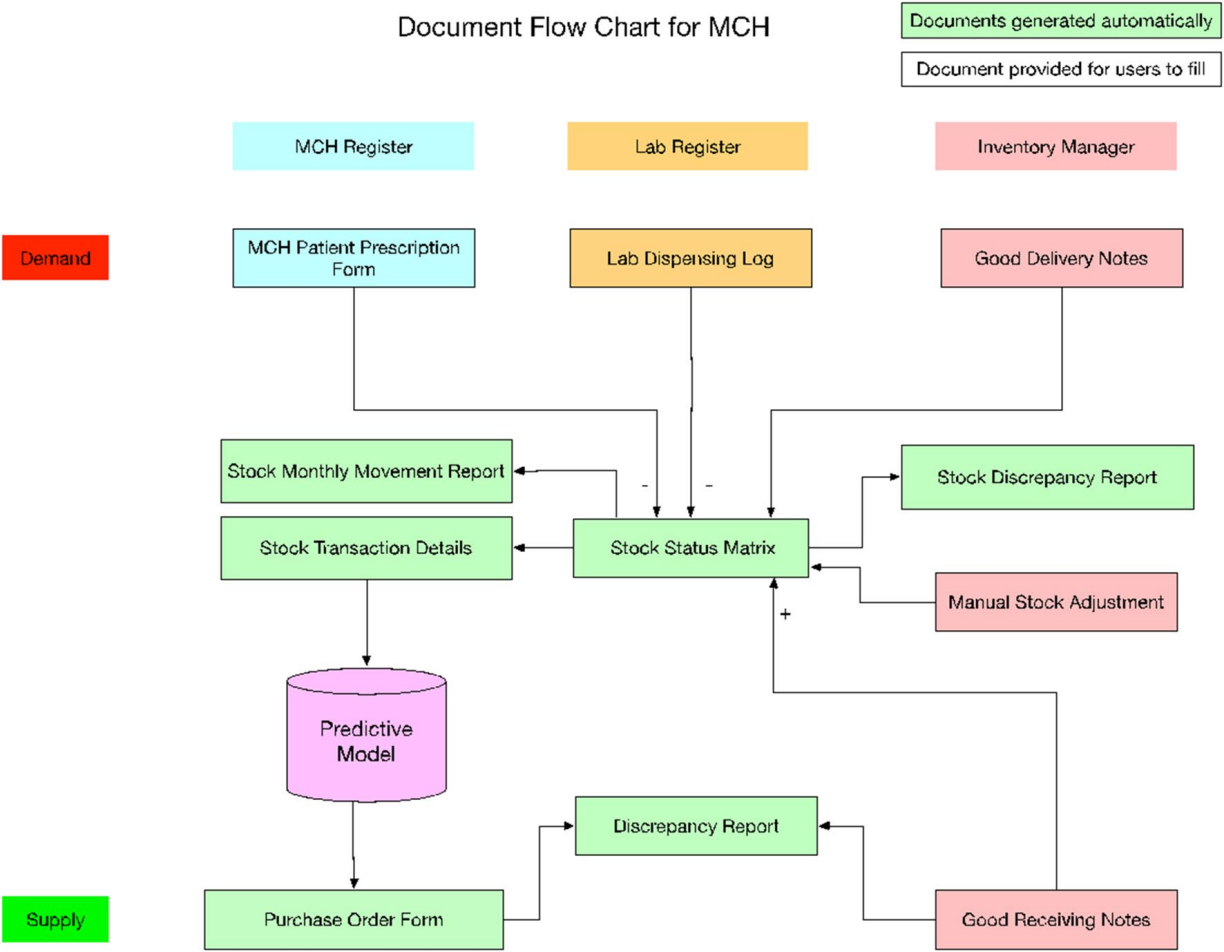
Document flow for MCH

With redesigned forms, inventory levels were updated automatically upon submission of *patient prescription form*, *lab dispensing log*, and *good delivery note* (Fig. 2). Stock reports such as inventory levels, monthly movements, transaction details, and discrepancy reports, were generated automatically. The predictive model learns from consumption and supply data to forecast and generate orders for the national store.

## Results

### System testing and deployment

We deployed two sets of equipment during our 2nd visit on Sept. 23–29, 2018:

- Mukono Health Center IV: laptop, router, wi-fi extender, 8 tablets.
- Kojja Health Center IV: laptop, router, wi-fi extender, 5 tablets.

Main store, lab and MCH units—one tablet for each, with an extra for backup. Both facilities encountered wi-fi signal strength issues. At Mukono, the delivery room is far from the nearest extender. There was no extra outlet in the MCH unit to install another wi-fi extender. At Kojja, lab and MCH are too far from the router that was installed in the server room powered by solar panel and located in a different building. We installed powered access point with wires extended to the MCH unit.

We revisited those two facilities five months later in March 2019.

### Summary of data collected from system deployment

We implemented this system in two health facilities in Uganda: Kojja and Mukono HC IV over 6 months. In Mukono, a larger site with more patients, patient admission forms were mostly filled using the new system, leaving us only basic information such as name, age, and village. In Kojja, more forms were filled but counts are lower than expected due to an unstable network caused by construction and power outages. Overview of data collected from the two facilities over 6 months is listed in Table 3.

**Table 3:** Data overview

Department	Forms	Kojja HC IV	Mukono HC IV
MCH	Patient admission	1275	4417
	Patient diagnosis	104	6
	Patient prescription	906	43
	Patient delivery	39	0
	Patient lab tests	252	11
Lab	Lab dispensing report	3	0
	Lab activity report	16	0
Main store	Main store receiving form	9	0
	Main store distribution form	0	1

### Predictive model

To improve the demand forecasting of medical supplies, predictive models were developed in this study based on the data collected from the system. Due to the time constraint of data collection, not all the medical products and their trend can be captured in the short data collection time frame. Health products were classified into three types based on the amount of past data, by which we will select appropriate prediction methods.

- *Type 1* Demand forecasting is not applicable due to the lack of previous consumption data
- *Type 2* Demand forecasting for immediate future is possible with limited consumption data
- *Type 3* Demand forecasting for an extended period of time is possible with sufficient consumption data

Three predictive models were developed for the three different types of health products based on the amount of available previous consumption data. For type 1 products, the minimum required amount and budget limit are directly used without actual past data to determine order quantities. Table 4 presents the health products and their predicted demands determined by aggregating responses from experts without actual data.

**Table 4:** Type 1 item prediction result

Item ID	Item name	Actual demand	Predictive demand	Ven scale
1	Artemether/Lumefantrine 120 mg tablet	22	30	Vital
7	Determine HIV Screening tests	30	30	
10	Malaria Rapid Diagnostic tests	3	10	
21	Nevirapine (NVP) 50 mg	3	10	
22	Cotrimoxazole 960 mg tablet	30	30	Vital
28	Ceftriaxone 1 g Injection	1	10	Vital
46	Iron	270	300	Essential
100	Pregnancy test strips 50 strips	100	100	
346	Erythromycin tablets bp 250 mg	120	120	Vital
353	Etonogestrel 150 mg implant (implanon)	1	10	Vital
357	Lamivudine, Zidovudine and Nevirapine tablets	1470	1500	
360	Efavirez, Lamuvidine, Torofoir, Disoproxil, Fumarate 600/300/300 mg	1750	1800	
365	Multivitamin tablets	120	120	Necessary
339–1	Doxycycline capsules 100 mg	80	80	Vital
339–2	CANNULA I.V, 20G. 0.9MM	36	40	Essential

For type 2 health products, average monthly consumptions are used to forecast the demand for the next month as the data accumulated possess information needed to derive average demand. It was observed that only 5 items in the collected data have more than 1-month demand. Table 5 shows the type 2 products and their predicted demands determined by averaging the consumption data over a certain period (e.g., 3–4 months).

**Table 5:** Type 2 item prediction result

Item ID	Item name	Actual demand	Predictive demand	Ven scale
3	Co-tromoxazole 480 mg tablet	250	285	Vital
15	Zidovudine/lamivudine/nevirapine	215	210	
44	Folic Acid	600	620	Essential
347	Amoxicilin capsules	25	30	Vital
349	Metronidazole tablets	60	75	Vital

For type 3 products, double exponential smoothing method were applied to predict the future demands as the consumption information is sufficient to capture the demand fluctuation as well. Double exponential smoothing is a time series forecasting method for univariate data that can be extended to support data with a systematic trend or seasonal component. To obtain the best double exponential smoothing model in time series prediction, trial and error tests were conducted to select the combinations of smoothing constants (alpha value and gamma value) with least mean absolute percentage error (MAPE) and median absolute deviation (MAD) in model prediction.

**Fig. 3:**
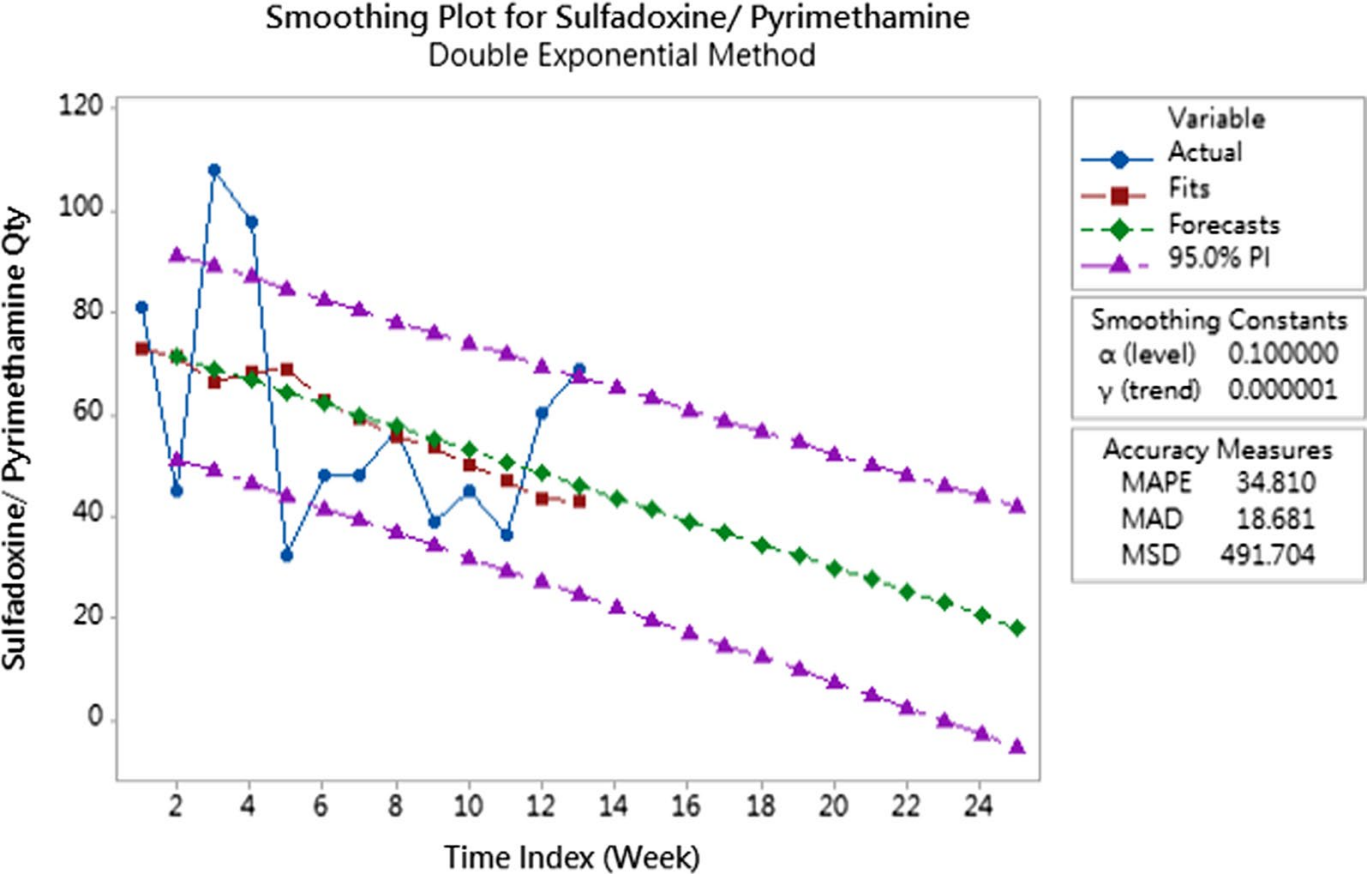
Demand prediction for sulfadoxine/pyrimethamine tablet

**Fig. 4:**
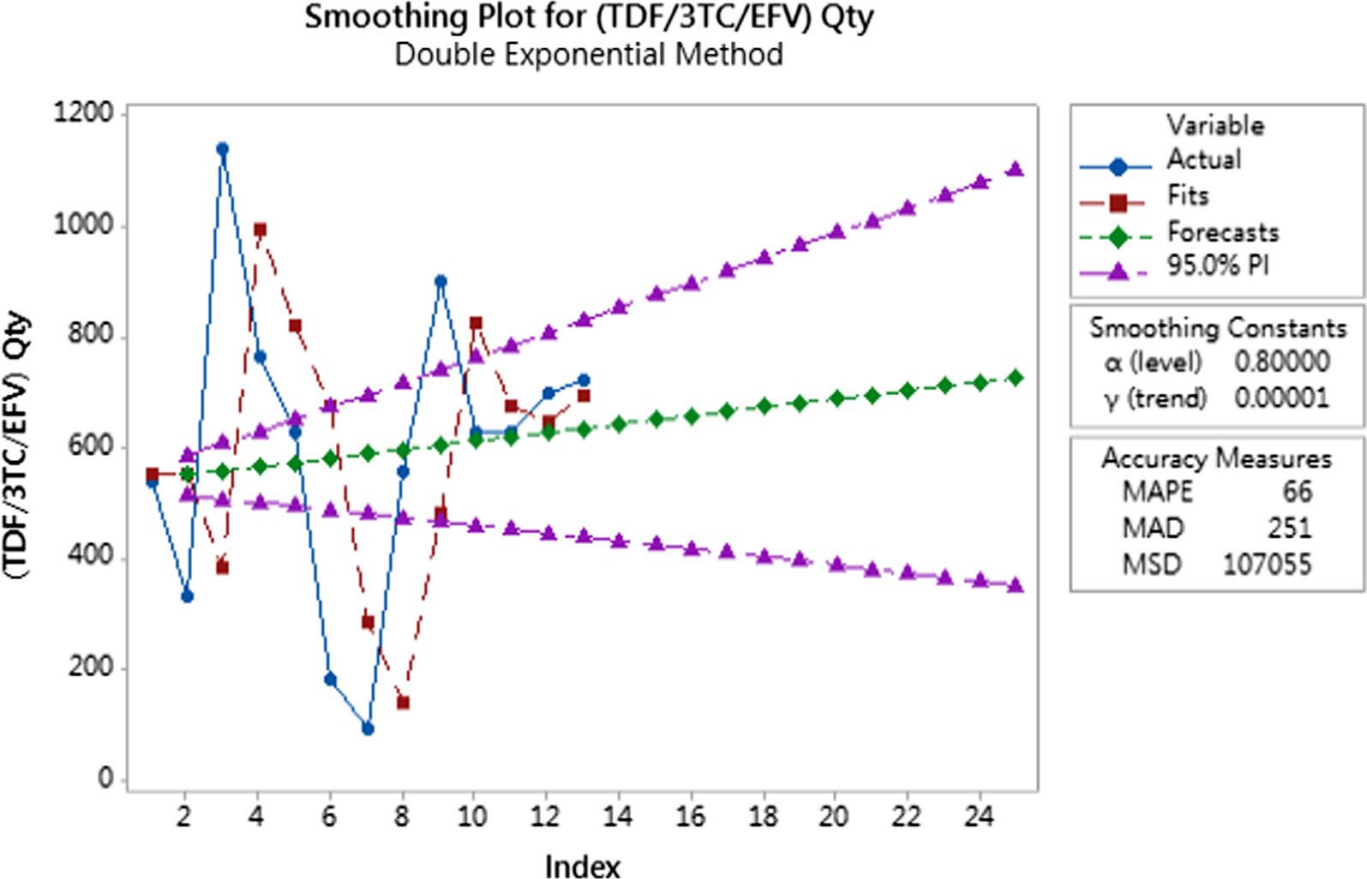
Demand prediction for tenofovir/lamivudine/efavirenz tablet

**Fig. 5:**
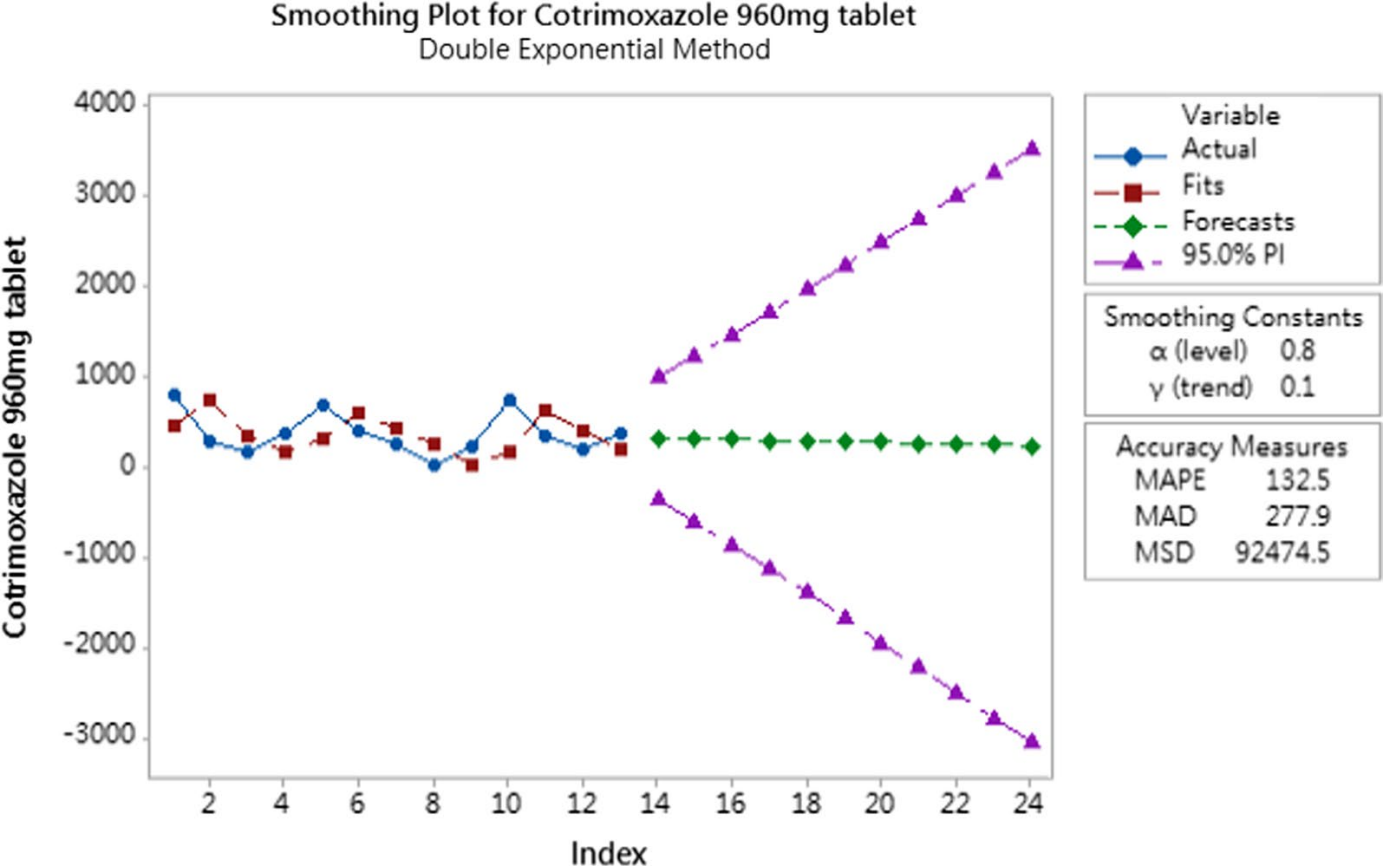
Demand prediction for cotrimoxazole tablet

From the collected data, it was discovered that only three items (Sulfadoxine/Pyrimethamine tablet, Tenofovir/Lamivudine/Efavirenz tablet and Cotrimoxazole tablet) have sufficient information in applying double exponential smoothing method for next three to six months demand prediction. The preliminary trends of demand prediction can be shown on Figs. 3, 4 and 5.

The demand pattern of Sulfadoxine/Pyrimethamine tablets were found to have less apparent trend compared to the other two products. From the fitting line generated by the double exponential smoothing method, the model could not capture the abrupt quantity increase or decrease very well. In this perspective, we can infer a double exponential smoothing model works better in the prediction of the items with apparent trend and less demand fluctuation. Despite the model not being able to capture the demand pattern effectively, this model has relatively good accuracy measures compared to another smoothing constant combination. The demand forecast trend developed has shown that the demand quantity of Sulfadoxine/Pyrimethamine tablets will be decreased in the next three months. Different from Sulfadoxine/Pyrimethamine, the fitting lines of the other two supplies (Tenofovir/Lamivudine/Efavirenz tablet and Cotrimoxazole tablet) have better performance in capturing the future demand trend because the consumption data have apparent trend. The prediction results show that the demand of Tenofovir/Lamivudine/Efavirenz in the next three months will increase slightly and that of Cotrimoxazole will decrease slightly.

In summary, the forecasted results indicated that, based on the data collected by the implemented system, the future demands can be forecasted according to the type of demand. However, the proposed approach has some limitations. First, the accuracy of forecasted demands largely depends on the amount of consumption data collected by the system. Additionally, the suggestion system for procurement based on the forecasted demand in real time has not been addressed. Thus, in order to increase the accuracy of prediction, we will keep track of demand changes and utilize the forecasted demands to provide more sophisticated order suggestions.

## Discussion

The current Uganda’s health management information system (HMIS), Uganda District Health Management Information Software System version 2 (DHIS2) focuses on morbidity reporting and covers data collected from health units to the district and national levels [8, 8, 8]. It was observed that the installation of DHIS2 did not result in improved utilization of health reports generated at district level [8]. It’s needed to implement am information system at each health facility to guarantee high levels of service and minimize stockouts [25]. Without a proper information system, health facilities continue to submit paper-based forms, challenged with inaccurate and inconsistent records. This directly affects the quality of reports submitted to the districts and eventually to the MoH [8] for resource allocation and procurement planning. The proposed E+TRA Health was developed to address those gaps in the information flow in the last mile. It has shown to successfully digitalize both supply and demand data from main store, lab and MCH to identify and forecast overstock and understock life-saving commodities for women and children. The proposed E+TRA Health system, implemented with tablets and local network structure, shows the capability of filling the gap where HMIS tools not rolled down to all local health facilities.

In addition, very few studies connect EMR with inventory management [29]. For MCH demand forecast, medications and supplies needed for antenatal care and on the day of delivery are predictable with useable and accurate EMR. E+TRA Health demonstrates the feasibility to use data from EMR to estimate the needs of 13 MCH essential supplies to inform supply chain management, enabling the coordination between MCH unit, clinical laboratory, and the main store for better care [30, 31].

This article presents a demand sensing and digital tracking system that was piloted to analyze information flow and address critical paths of supplies associated with MCH in the Ugandan health system. Three datasets were redesigned and digitized: (1) MCH Unit: *Patient Admission*, *Diagnosis*, *Delivery*, *Prescription*, *Lab Results*; (2) Main store: *Goods Delivery*, *Receiving*; and (3) Laboratory: *Commodity Dispensing*, *Daily Activity Report*. Functions such as item coding, generation of monthly reports directly from the system, and generation of automatically suggested procurement orders were introduced. We implemented this system in two pilot sites at Kojja and Mukono Healthcare Center IV facilities in Uganda. Lists of supplies needed throughout the lifecycle of MCH continuum of care are studied based on 6 months data.

### Key lessons

Key lessons learnt and challenges during this pilot study are summarized in the following categories: healthcare workflow, stakeholder engagement process and local infrastructure and capacities.

#### Healthcare workflow

The district medical store receives health products (medicines, supplies and laboratory reagents) every 2 months from National Medical Store (NMS) according to a predefined schedule for the six cycles of the year and stores them temporarily, for few days until they are distributed to health facilities in with 3 ~ 4 days after receipt. The products delivered to the district medical store are already earmarked to each of the health facilities. A private contractor, called 3-ways, picks the products from the district medical store and delivers them to each of the health facilities in the district. At the facility, the storekeepers/managers receive the products and accompanying documents (delivery notes and invoices). The physical type, count, expiry dates and other physical features are checked against the delivery notes and invoice. If there is any discrepancy, discrepancy form is filled out and signed by all the parties concerned. The delivery of the products happens in the presence of a representative from council and health unit management committee. In general, products are short delivered compared to cyclic orders both in type and quantity. In 2018, more than 80% of Health Centers (HCs) and hospitals were stocked out of one or more health commodities. [41].

We observed multiple issues in the overall process:

1. The storekeepers/lab managers order just based on their experience and many times that estimation is incorrect resulting in overstock or understock.
2. Once received, there is no mechanism to receive the supplies for understock items until the next cycle is due.
3. It takes multiple days for the storekeeper to update all the stock cards.
4. There is no existing mechanism to communicate among health facilities below level IV to exchange overstock supplies.
5. A tool for tracking unused money is highly preferred. Each of the health facilities has its own allocated money that is given to NMS at the beginning of the financial year. But when they fail to provide any commodity, it does not show the amount of unused money to the district offices or health facilities. The importance of a tool that can track expenditure and compare with the initial budget to calculate the balance remaining at the time of delivery of products was recommended.
6. There is a big gap in the annual budget for medicines and supplies (does not include laboratory reagents and supplies). In Kojja Health center IV, the budget was 52 million Shilling/year which is roughly 38% of the actual need. In this scenario quantification is one of the biggest challenges. The storekeeper needs to make a judgment call regarding how much to order for each essential commodity within this limited budget.

#### Stakeholder engagement process

Securing early buy-in from MOH is very important. The Ministry has a number of bureaucratic channels and levels through which the necessary buy-in can be obtained. The earliest level for buy-in is at the ‘user’ department or unit who are the potential users of the innovation. Typically, a department to which the proposed innovation speaks. The department assigns a focal person who should understand how the innovation works. This focal point is supposed to handhold the innovation team to navigate the next level of buy-in. This involves a series of pitches to the Ministry's Technical Working Groups (TWGs).

1. The first pitch at a user (beneficiary) technical department level TWG, in our case the MCH TWG. The TWG makes an endorsement indicating ownership of the innovation and recommends to the next level of approval, which is e-Health TWG.
2. E-Health TWG coordinates and approves all innovations that are piloted within the MOH infrastructure and system. The e-Health TWG vets the proposed innovation and how the innovation will support the work of the user technical department. This TWG thereafter makes approval for innovation pilot and testing within the Ministry.

While MOH has been championing for a migration from paper-based system to electronic system, there is still a long way to go. The Ministry has a number of e-health systems that have been piloted, and some scaled. However, there is still a large reliance on paper-based systems to collect and report routine data including HMIS data.

The health workers at both Kojja and Mukono where the innovation was piloted exhibited large interests and enthusiasm. The use of portable tablets was new to them. The health workers had been accustomed to collecting routine data using paper-based HMIS forms.

Incentives in the form of allowances to the health workers and volunteer research assistants were key for data collection to proceed. Health workers continue to operate with paper-based forms as required by the MoH, so the allowances are necessary for them to take on this additional task.

#### Local infrastructure and capacities

E+TRA Health is designed for intermittent internet connectivity. For daily operation, it operates without the internet. When online, the system provides additional features such as backups, system updates, and remote diagnosis/troubleshooting. As infrastructure improves and internet connectivity becomes more accessible and reliable, E+TRA Health can provide real-time monitoring and visibility of inventory levels, improving coordination and strategic planning among facilities locally, regionally and nationally.

We faced challenges of setting up intranet networks within both facilities. The units where services are delivered, MCH, laboratory, main store and dispensary, are not in close proximity, spreading over multiple isolated buildings that are located apart. Setting up an effective intranet was a challenge right from the start of the pilot with limited local supplies, technical support and capacities.

In addition, power was unreliable at both Kojja and Mukono, frequently suffering from outages. During the time of our pilot, there was a power line replacement that took about 3 months. Unscheduled two-to-four-hour power outage each day during our pilot imposed further challenge in wi-fi coverage. For future design, backup battery for all IT equipment/devices, especially intranet wi-fi related devices, will be important to include. In addition, adequate budget and understanding local capacities for installing and sustaining/maintaining IT system and internet/intranet network are also critical to consider for making long-term systemic change.

### Limitation and future works

The accuracy of forecasted demands largely depends on the amount of consumption data collected by the system. However, this proposed system was originally designed and focused on MCH only as a proof-of concept of integrating supplies chain management systems with demand information collected from health systems. We observed that some supplies for MCH are also shared among other departments. The consumption data are not complete unless we implement E+TRA Health in all care units in the facility. Additional, due to the short duration (6 months) of this pilot and small amount of data collected, only basic prediction models were developed/used. This system can be improved by extending the scope of the study to cover all care units with a longer period.

## Conclusions

We designed a cloud-based, multi-platform, offline-compatible healthcare supply chain management system tailored for MCH. It is capable of triangulating supply and demand data from three different departments (main store, lab, and MCH) to forecast and generate orders automatically to meet patient demands. It is capable of generating reports required by MOH in real time compared to one-week lead-time using paper-based systems. This prompts frontline stakeholders to generate efficient, reliable and sustainable strategic healthcare plans with real time data. This system improves patient outcomes through better commodity availability by sensing true patient demands. Key lessons learnt and challenges during implementation about (1) healthcare workflow, (2) stakeholder engagement process, (3) local infrastructure and capacities, are discussed to inform future work.

## Supplementary Information

**Additional file 1: Fig. S1.** Example mobile device form. **Fig. S2**. Generated HMIS printable form. **Fig. S3**. Generated plots. **Fig. S4**. Full history of transactions of each commodity. **Fig. S5**. HMIS Forms: Integrated Antenatal Register and Integrated Maternity Register.

## Data Availability

The datasets used and/or analyzed during the current study available from the corresponding author on reasonable request.

## Abbreviations

E+TRA Health: Electronic tracking system for healthcare commodities
MCH: Maternal child health
MOH: Ministry of health
SCM: Healthcare supply chain management
EMR: Electronic medical record
UN: United Nations
VHTs: Village health teams
HIS: Health information system
HMIS: Health management information system
DHO: District health office
HIV: Human immunodeficiency virus
IKT: Integrated knowledge translation
CIHR: Canadian Institute of Health Research
RAN: Resilient Africa network
TWGs: Ministry's technical working groups
OPD: Out-patient department
MAPE: Mean absolute percentage error
MAD: Median absolute deviation
NRHs: National referral Hospitals
RRHs: Regional referral hospitals
HC: Health center
DHIS2: District health management information software system version 2

## Footnotes

1 https://gcgh.grandchallenges.org/challenge/health-systems-strengthening-ensuring-effective-health-supply-chains-round-19.

## References

[1] Say L, Chou D, Gemmill A, Tunçalp Ö, Moller AB, Daniels J, Gülmezoglu AM, Temmerman M, Alkema L (2014). Global causes of maternal death: a WHO systematic analysis. Lancet Glob Health.

[2] Ronsmans C, Graham WJ (2006). Maternal mortality: who, when, where, and why. Lancet.

[3] Tort J, Rozenberg P, Traoré M, Fournier P, Dumont A (2015). Factors associated with postpartum hemorrhage maternal death in referral hospitals in Senegal and Mali: a cross-sectional epidemiological survey. BMC Pregnancy Childbirth.

[4] Mbonye AK (2001). Risk factors associated with maternal deaths in health units in Uganda. Afr J Reprod Health.

[5] Mbonye AK, Mutabazi MG, Asimwe JB, Sentumbwe O, Kabarangira J, Nanda G, Orinda V (2007). Declining maternal mortality ratio in Uganda: priority interventions to achieve the Millennium Development Goal. Int J Gynecol Obstet.

[6] Jonathan HG, Stoltenberg RH (2012). UN commission on life-saving commodities for women and children.

[7] MOH. Health sector strategic & investment plan: promoting people’s health to enhance socio-economic development title. http://www.health.go.ug/docs/HSSIP10.pdf. Accessed 15 Dec 2020.

[8] Kiberu VM, Matovu JK, Makumbi F, Kyozira C, Mukooyo E, Wanyenze RK (2014). Strengthening district-based health reporting through the district health management information software system: the Ugandan experience. BMC Med Inform Decis Mak.

[9] Landén EN, MHealth systems, Transformations in Work and Implications for Sustainability. In: PhD thesis. University of Oslo. 2019 Jan. https://www.mn.uio.no/ifi/english/research/networks/hisp/research-library/thesis/esther.pdf. Access 15 Dec 2020.

[10] Gladwin J, Dixon RA, Wilson TD (2003). Implementing a new health management information system in Uganda. Health Policy Plan.

[11] Kintu P, Nanyunja M, Nzabanita A, Magoola R (2005). Development of HMIS in poor countries: Uganda as a case study. Health policy and development..

[12] Jawhari B, Ludwick D, Keenan L, Zakus D, Hayward R (2016). Benefits and challenges of EMR implementations in low resource settings: a state-of-the-art review. BMC Med Inform Decis Mak.

[13] Boonstra A, Versluis A, Vos JF (2014). Implementing electronic health records in hospitals: a systematic literature review. BMC Health Serv Res.

[14] Fraser H, Biondich P, Moodley D, Choi S, Mamlin B, Szolovits P (2005). Implementing electronic medical record systems in developing countries. J Innov Health Inf.

[15] Williams F, Boren S (2008). The role of the electronic medical record (EMR) in care delivery development in developing countries: a systematic review. J Innov Health Inf.

[16] Chib A, van Velthoven MH, Car J (2015). mHealth adoption in low-resource environments: a review of the use of mobile healthcare in developing countries. J Health Commun.

[17] Beratarrechea A, Lee AG, Willner JM, Jahangir E, Ciapponi A, Rubinstein A (2014). The impact of mobile health interventions on chronic disease outcomes in developing countries: a systematic review. Telemed e-Health.

[18] Antao V, Pinheiro G (2015). Surveillance for occupational respiratory diseases in developing countries. Semin Respir Crit Care Med.

[19] Nsubuga P, Nwanyanwu O, Nkengasong JN, Mukanga D, Trostle M (2010). Strengthening public health surveillance and response using the health systems strengthening agenda in developing countries. BMC Public Health.

[20] Haskew J, Turner K, Rø G, Ho A, Kimanga D, Sharif S (2015). Stage of HIV presentation at initial clinic visit following a community-based HIV testing campaign in rural Kenya. BMC Public Health.

[21] Blaya JA, Cohen T, Rodríguez P, Kim J, Fraser HS (2009). Personal digital assistants to collect tuberculosis bacteriology data in Peru reduce delays, errors, and workload, and are acceptable to users: cluster randomized controlled trial. Int J Infect Dis.

[22] Fraser HS, Jazayeri D, Nevil P, Karacaoglu Y, Farmer PE, Lyon E, Fawzi MK, Leandre F, Choi SS, Mukherjee JS (2004). An information system and medical record to support HIV treatment in rural Haiti. BMJ.

[23] Were MC, Shen C, Bwana M, Emenyonu N, Musinguzi N, Nkuyahaga F, Kembabazi A, Tierney WM (2010). Creation and evaluation of EMR-based paper clinical summaries to support HIV-care in Uganda, Africa. Int J Med Inf.

[24] Tweya H, Feldacker C, Haddad LB, Munthali C, Bwanali M, Speight C, Kachere LG, Tembo P, Phiri S (2017). Integrating family planning services into HIV care: use of a point-of-care electronic medical record system in Lilongwe, Malawi. Glob Health Action.

[25] Yadav P (2015). Health product supply chains in developing countries: diagnosis of the root causes of underperformance and an agenda for reform. Health Syst Reform.

[26] Olok GT, Yagos WO, Ovuga E (2015). Knowledge and attitudes of doctors towards e-health use in healthcare delivery in government and private hospitals in Northern Uganda: a cross-sectional study. BMC Med Inform Decis Mak.

[27] Liang L, Wiens MO, Lubega P, Spillman I, Mugisha S (2018). A locally developed electronic health platform in Uganda: development and implementation of Stre@ mline. JMIR Form Res.

[28] Haskew J, Rø G, Saito K, Turner K, Odhiambo G, Wamae A, Sharif S, Sugishita T (2015). Implementation of a cloud-based electronic medical record for maternal and child health in rural Kenya. Int J Med Inf.

[29] Kiberu VM, Mars M, Scott RE (2017). Barriers and opportunities to implementation of sustainable e-Health programmes in Uganda: a literature review. Afr J Prim Health Care Fam Med.

[30] Tukamuhabwa B, Stevenson M, Busby J. Supply chain resilience in a developing country context: a case study on the interconnectedness of threats, strategies and outcomes. Suppl Chain Manag Int J. 2017 Sep 11.

[31] Madinah N (2016). Challenges and barriers to the health service delivery system in Uganda. IOSR J Nurs Health Sci.

[32] Graham ID, Kothari A, McCutcheon C (2018). Moving knowledge into action for more effective practice, programmes and policy: protocol for a research programme on integrated knowledge translation. Implement Sci.

[33] Kreindler SA (2018). Advancing the evaluation of integrated knowledge translation. Health Res policy Syst.

[34] Kothari A, Wathen CN (2013). A critical second look at integrated knowledge translation. Health Policy.

[35] Walton K, Ambrose T, Annis A, Ma DW, Haines J (2018). Putting family into family-based obesity prevention: enhancing participant engagement through a novel integrated knowledge translation strategy. BMC Med Res Methodol.

[36] McIsaac JL, Penney TL, Storey KE, Sigfridson L, Cunningham J, Kuhle S, Kirk SF (2018). Integrated knowledge translation in population health intervention research: a case study of implementation and outcomes from a school-based project. Health Res Policy Syst.

[37] Gagliardi AR, Dobrow MJ (2016). Identifying the conditions needed for integrated knowledge translation (IKT) in health care organizations: qualitative interviews with researchers and research users. BMC Health Serv Res.

[38] Roberge-Dao J, Yardley B, Menon A, Halle MC, Maman J, Ahmed S, Thomas A (2019). A mixed-methods approach to understanding partnership experiences and outcomes of projects from an integrated knowledge translation funding model in rehabilitation. BMC Health Serv Res.

[39] Web Technology Surveys. Usage of server-side programming languages for websites. https://w3techs.com/technologies/overview/programming_language. 15 Dec 2020.

[40] Asay M. Php and perl crashing the enterprise party. Feb 2010. https://www.cnet.com/news/php-and-perl-crashing-the-enterprise-party/. 15 Dec 2020.

[41] Lugada E, Komakech H, Ochola I (2022). Health supply chain system in Uganda: current issues, structure, performance, and implications for systems strengthening. J of Pharm Policy and Pract.

