# Supplementary material for: Demand sensing and digital tracking for maternal child health (MCH) in Uganda: a pilot study for ‘E+TRA health’
Source: BMC Med Inform Decis Mak. 2022 Sep 12;22:239. doi: 10.1186/s12911-022-01982-8 (PMC9469598; doi:10.1186/s12911-022-01982-8)
Supplement: Supplementary file 1 — Additional file 1: Fig. S1. Example mobile device form. Fig. S2. Generated HMIS printable form. Fig. S3. Generated plots. Fig. S4. Full history of transactions of each commodity. Fig. S5. HMIS Forms: Integrated Antenatal Register and Integrated Maternity Register. [file 12911_2022_1982_MOESM1_ESM.docx]

**Appendix**


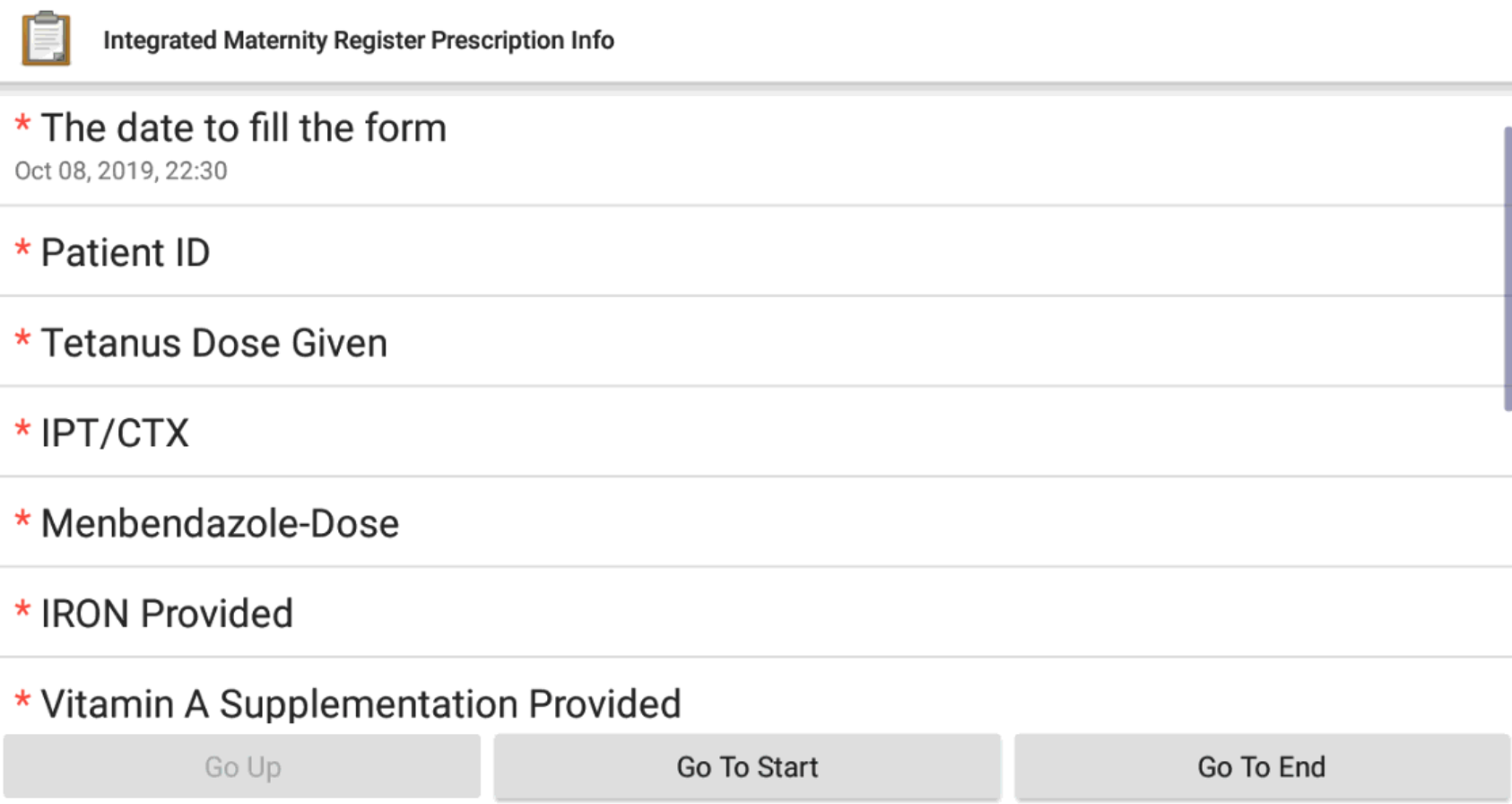


Supplementary Fig. 1 Example mobile device form


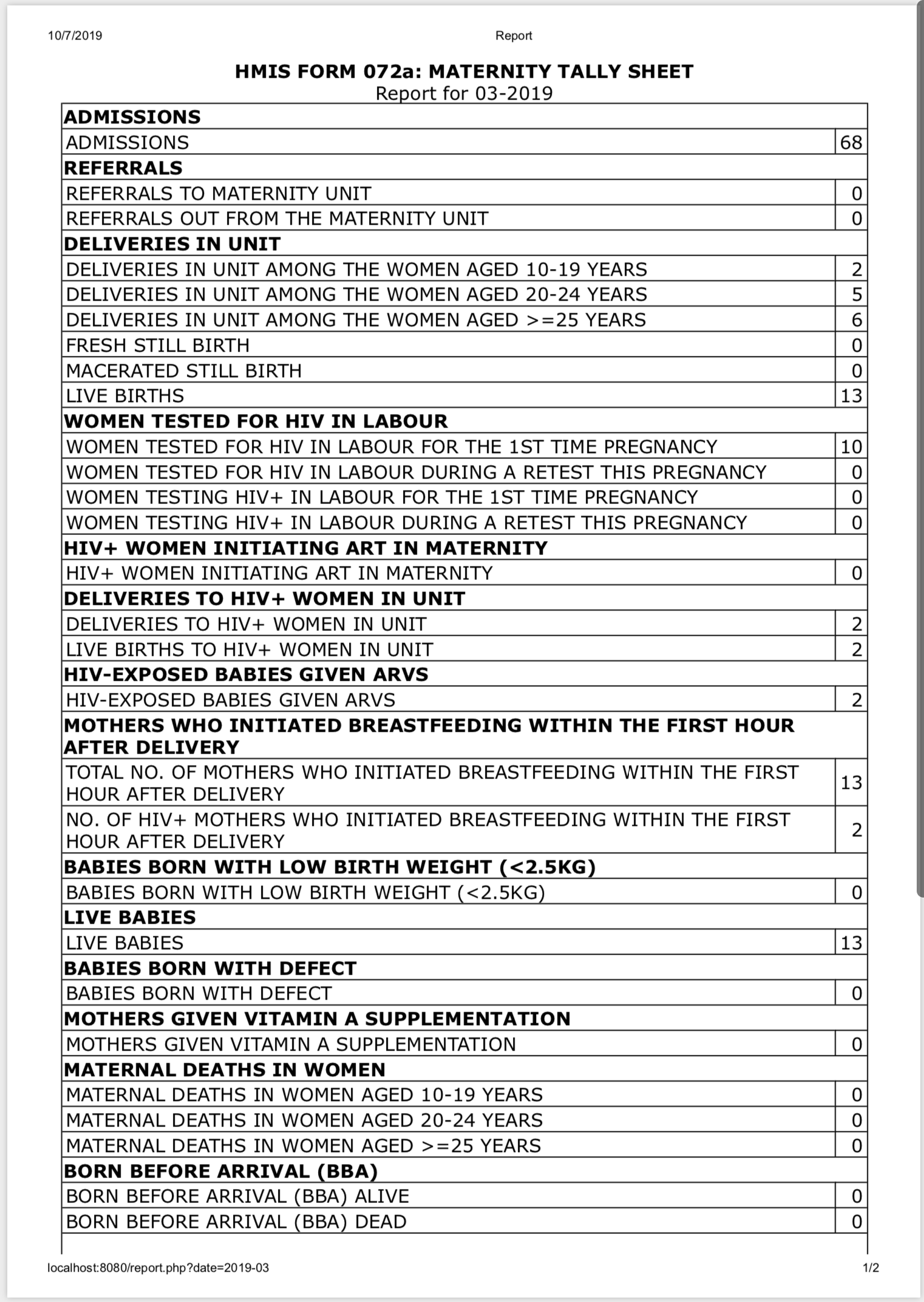


Supplementary Fig. 2 Generated HMIS printable form


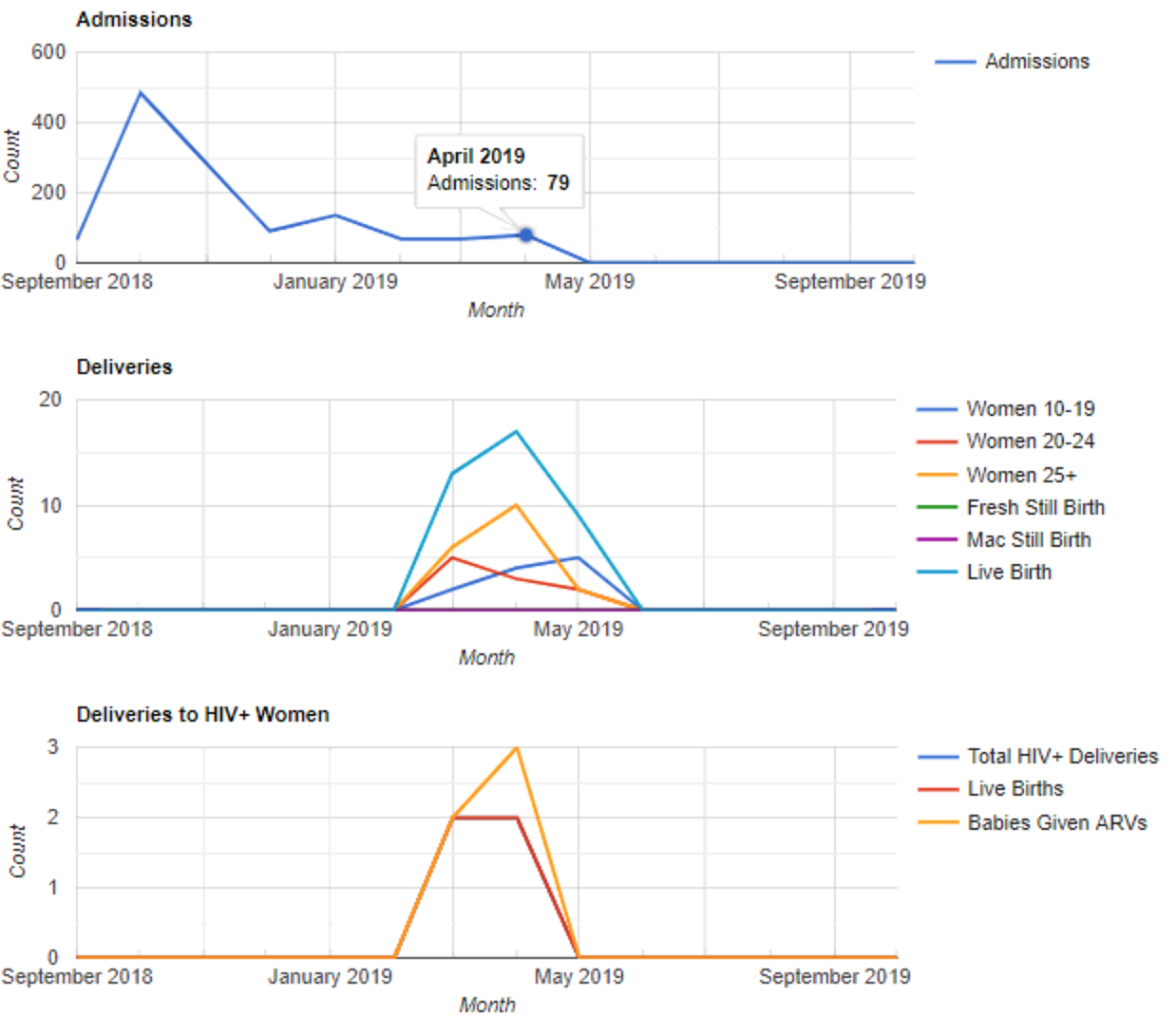


Supplementary Fig. 3 Generated plots

#
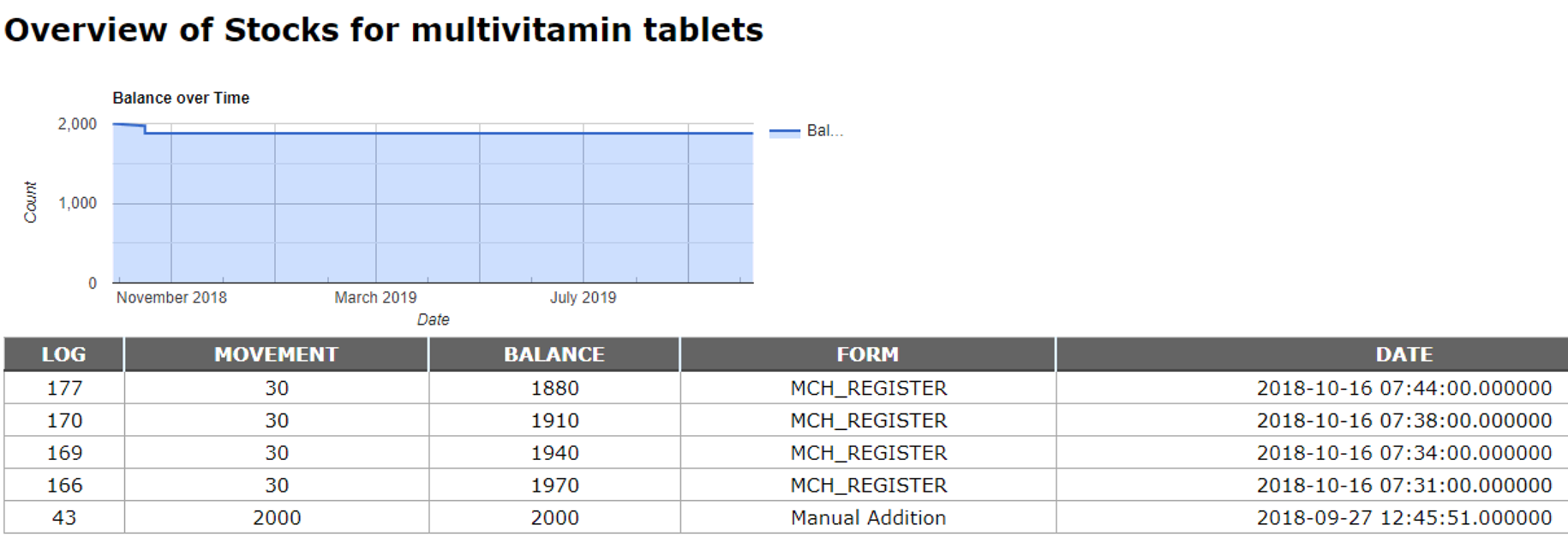


Supplementary Fig. 4 Full history of transactions of each commodity

#
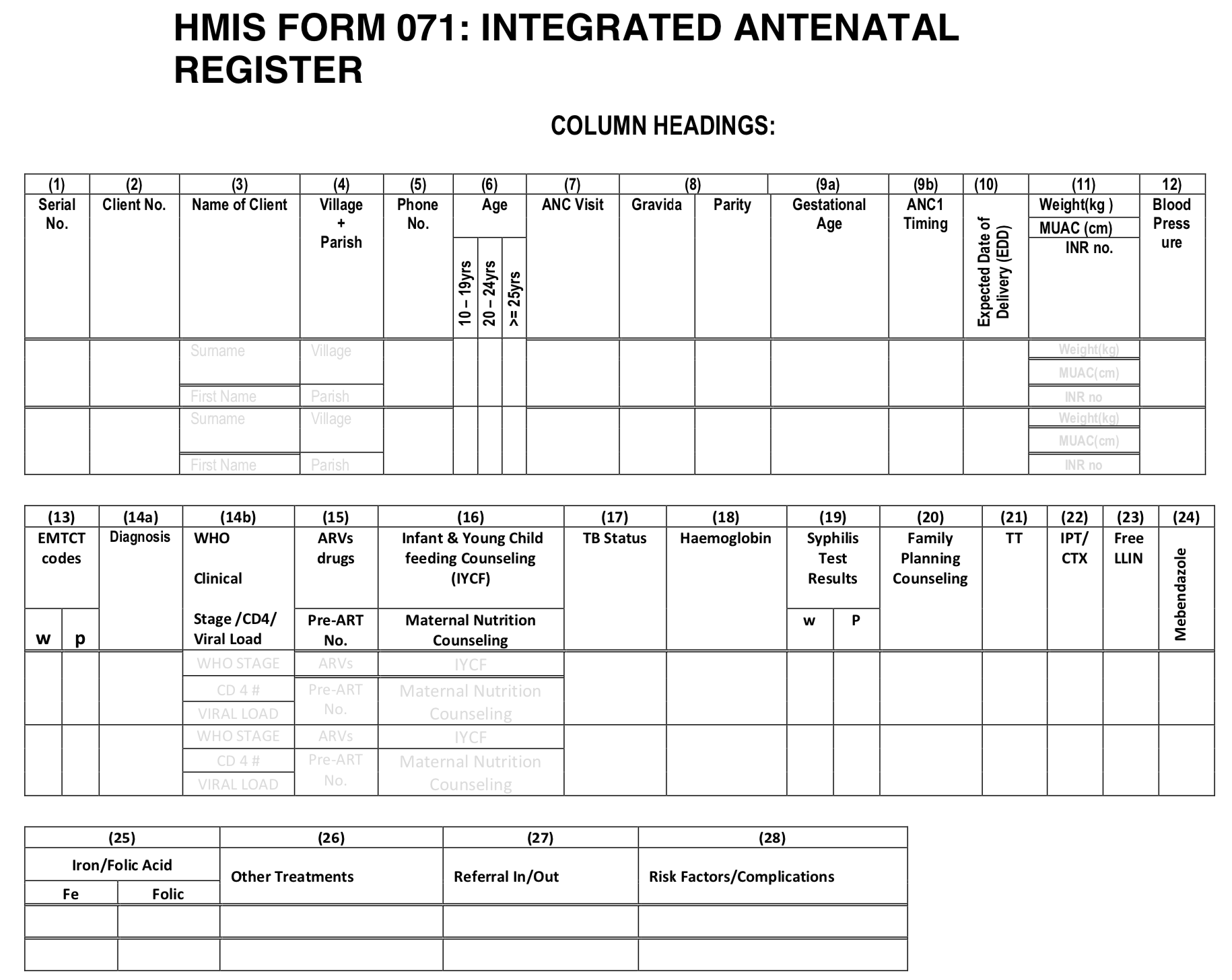

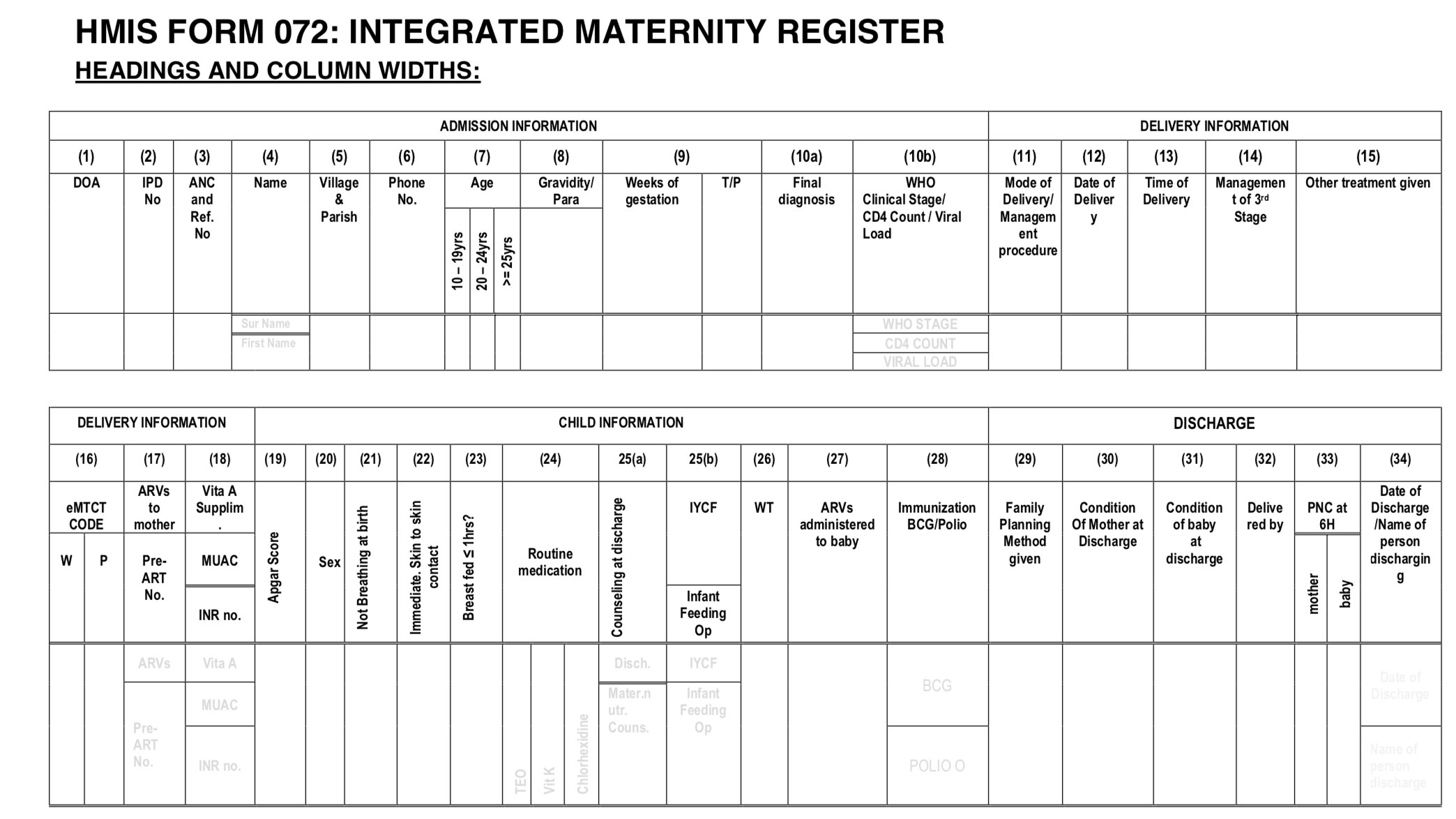


Supplementary Fig. 5. HMIS Forms: Integrated Antenatal Register and Integrated Maternity Register
